# Supplementary material for: Incommensurately Modulated Crystal Structure and Photoluminescence Properties of Eu2O3- and P2O5-Doped Ca2SiO4 Phosphor
Source: Materials (Basel). 2019 Dec 20;13(1):58. doi: 10.3390/ma13010058 (PMC6982207; doi:10.3390/ma13010058)
Supplement: Supplementary file 1 [file materials-13-00058-s001.pdf]

Supporting Information

Incommensurately Modulated Crystal Structure and Photoluminescence Properties of Eu_2O_3- and P_2O_5-doped Ca_2SiO_4 Phosphor

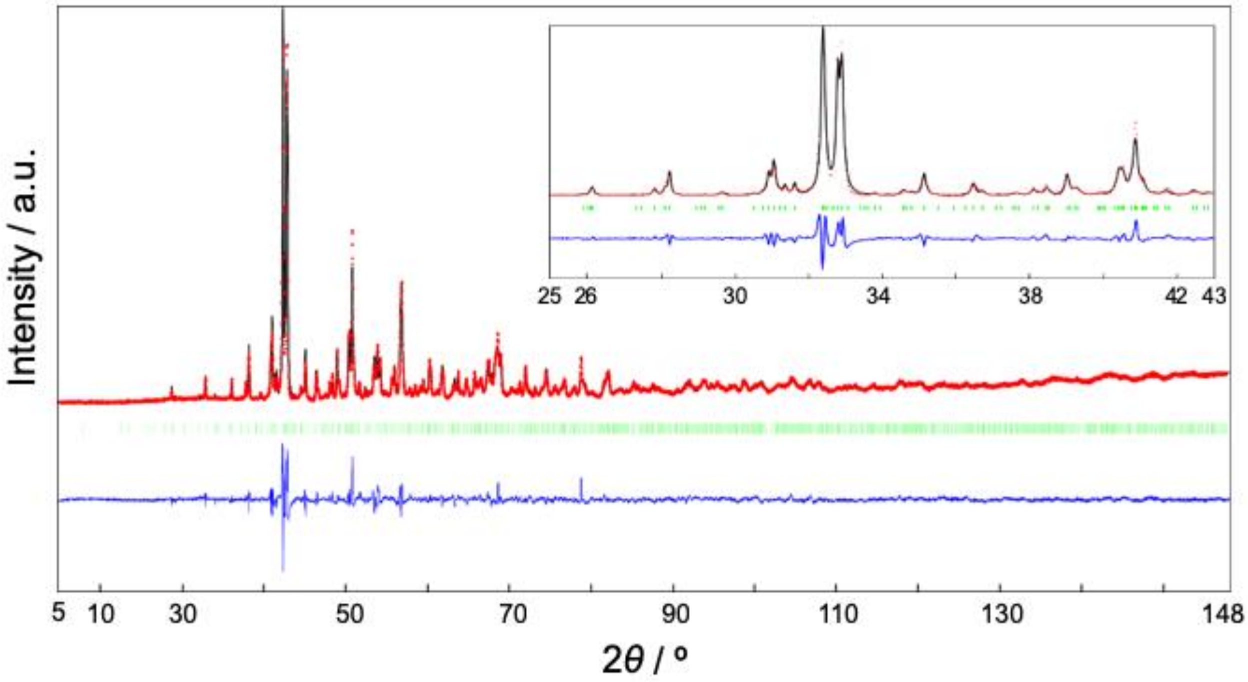

Figure S1. Comparison of the observed diffraction pattern of $(Ca_{1.950}Eu^{3+}_{0.013}\square_{0.037})(Si_{0.940}P_{0.060})O_4$ (symbol: +) with the corresponding calculated pattern (upper solid line). The difference curve is shown in the lower part of the diagram. Vertical bars indicate the positions of Bragg reflections.

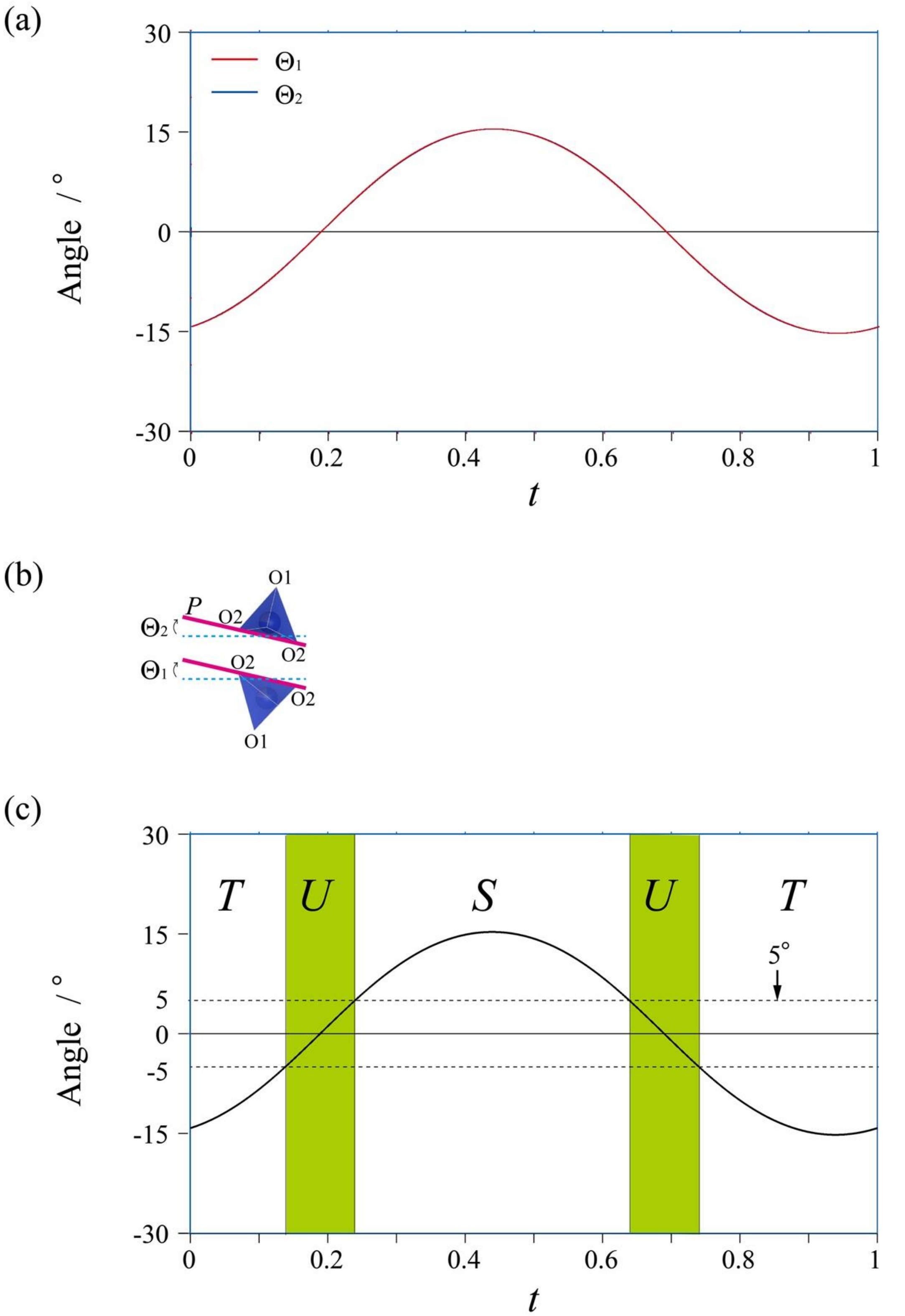

Figure S2. Tilting of the [MO_4] tetrahedron evaluated by the angle Θ. (**a**) The tilting angles Θ_1 (red line) and Θ_2 (blue line) with t. Because the differences between Θ_1 and Θ_2 in the whole regions of $0 \leq t \leq 1$ are within 0.1 degree, the corresponding two curves are almost overlapped with each other. (**b**) Two types of tetrahedra showing the tilting angles Θ_1 and Θ_2. (c) An average of the two angles $(\Theta_1 + \Theta_2)/2$ with t. The tilting angles Θ_1 and Θ_2 in (**a**) are defined for two types of tetrahedra as illustrated in (**b**). As Θ_1 and Θ_2 are slightly different, an average of the two angles, $\Theta = (\Theta_1 + \Theta_2)/2$ in (**c**), is used as the tilting angle in discussion for simplicity. Phase parameter t is defined by $t = x_4 - \mathbf{q} \times \mathbf{r}$, where x_4

is the fractional coordinate of the 4th direction in (3 + 1) dimensional superspace description, **q** is the modulation wavevector, and **r** is the positional vector.

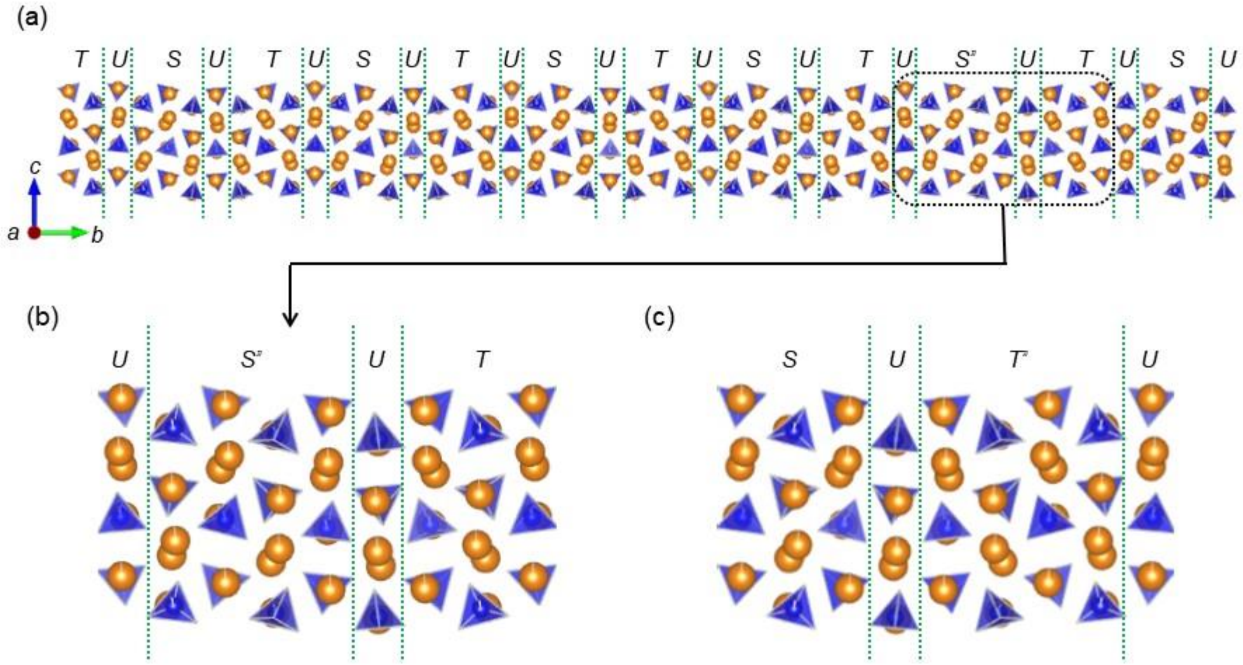

Figure S3. (**a**) Projection of a partial structure along the *a*-axis. (**b**) Enlargement of the structure, as indicated by an arrow in (**a**), showing the replacement of layer *S* with layer *S*′′. (**c**) An example of the replacement of layer *T* with layer *T*′′.
